# The Synthesis, Structure, and Dielectric Properties of a One-Dimensional Hydrogen-Bonded DL-α-Phenylglycine Supramolecular Crown-Ether-Based Inclusion Compound

**DOI:** 10.3390/molecules28227586

**Published:** 2023-11-14

**Authors:** Yang Liu, Hongzhi Hu, Huanhuan Qi, Meixia Lv, Zunqi Liu

**Affiliations:** 1 Chemistry and Chemical Engineering College, Xinjiang Agricultural University, Urumqi 830052, China; ly2021@xjau.edu.cn (Y.L.); lzq201685@163.com (H.H.); 15609905454@163.com (H.Q.); lvmeixia1105@163.com (M.L.); 2 Xinjiang Sub-Center, National Engineering Research Center of Novel Equipment for Polymer Processing, Urumqi 830052, China; 3 Xinjiang Key Laboratory of Agricultural Chemistry and Biomaterials, Urumqi 830052, China

**Keywords:** hydrogen bonding, molecular rocking, crown ether, metal complexes, dielectric anisotropy

## Abstract

A novel hydrogen-bonded supramolecular crown-ether-based inclusion compound, [(DL-α-Phenylglycine)(18-crown-6)]^+^[(CoCl_4_)_0.5_]^−^(**1**), was obtained via evaporation in a methanolic solution at room temperature using DL-α-phenylglycine, 18-crown-6, cobalt chloride (CoCl_2_), and hydrochloric acid. Its structure, thermal properties, and electrical properties were characterized via elemental analysis, single-crystal X-ray diffraction, variable-temperature infrared spectroscopy, thermogravimetric analysis, differential scanning calorimetry, and variable temperature–variable frequency dielectric constant testing. The compound was a monoclinic crystal system in the *C*_2_ space group at low temperature (100 K) and room temperature (293 K). Analysis of the single crystal structure showed that [(CoCl_4_)_0.5_]^−^ presented an edge-sharing ditetrahedral structure in the disordered state, while the protonated DL-α-phenylglycine molecule in the disordered state and intramolecular hydroxyl group (-OH) underwent dynamic rocking, causing a significant stretching motion of the O-H···Cl-type one-dimensional hydrogen bond chain. This resulted in dielectric anomalies in the three axes of the crystal, thus showing significant dielectric anisotropy.

## 1. Introduction

Given that supramolecular materials are predisposed to the formation of stable novel structures and molecular aggregates of specific functions, their diverse hybrid assemblies of functional units tend to form complex order–disorder states, which can give rise to anomalies in optical, electrical, thermal, magnetic, and other physical properties of the resulting materials [1,2,3,4,5,6,7,8,9,10,11,12,13,14,15,16]. Therefore, achieving the functional diversification and coupling of supramolecular material systems has become a research hotspot, especially in the fields of crystal engineering, nonlinear optics, and hybrid materials [17,18,19,20,21,22,23,24,25,26,27,28,29,30,31,32,33]. Host–guest crown-ether-based supramolecular structures, represented by organic amines and crown ethers, are prone to molecular rocking, proton transfers, and order–disorder transitions within the structural units of hybrid materials via intermolecular forces, hydrogen bonds, and other interactions. Therefore, these supramolecular structures have important applications in photoelectric conversion, storage devices, capacitors, and sensors [34,35,36,37,38,39,40,41,42,43,44]. The research group headed by Professor Xiong at Southeast University [45] successfully synthesized a high-temperature multi-axial host–guest inclusion ferroelectric material that undergoes phase transitions [(MeO-C_6_H_4_-NH_3_)(18-crown-6)][TFSA]. In this crystal structure, the 4-methoxyaniline cation is situated within the cavity of the 18-crown-6 molecule via hydrogen-bonding interactions to form a supramolecular structure, and the introduction of a larger TFSA anion causes a lower crystal symmetry at room temperature. This in turn causes an order–disorder phase transition from room temperature to low temperature, resulting in the symmetry breaking of its space group from *P*c to *P*_21_, which leads to a paraelectric–ferroelectric phase transition. Furthermore, it undergoes three phase transitions at 264 K, 311 K, and 415 K to form multi-axial ferroelectricity. The study demonstrated that this type of crystalline material has potential applications in sensing, driving, data storage, and flexible electronics. Crown ethers belong to the special planar macromolecular structure, which can easily form a large space in the crystal. Chiral amino acids can induce crystal growth into polar spatial groups. Meanwhile, these supramolecular compounds usually contain tetrahedral ions (CoCl_4_^2−^, BF_4_^−^, IO_4_^−^, etc.) acting as counter-anions. The combination of these three components may result in novel polar materials. In this study, DL-α-phenylglycine, 18-crown-6, cobalt chloride, and hydrochloric acid were selected as raw materials to synthesize a host–guest inclusion crown-ether-based supramolecular crystal [(DL-α-Phenylglycine)(18-crown-6)]^+^[(CoCl_4_)_0.5_]^−^(**1**) in a methanol solution, followed by the characterization and analysis of its structure and properties via single-crystal X-ray diffraction, variable-temperature infrared spectroscopy, powder X-ray diffraction, thermogravimetry, and dielectric tests.

## 2. Results and Discussion

### 2.1. Description of the Single-Crystal Structure of Compound ***1***

It can be seen from the SXRD results that compound **1** belongs to the monoclinic crystal system with a chiral space group C_2_ at low temperature (LT, 100 K) and room temperature (RT, 293 K). The lattice parameters at LT (100 K) were *a* = 23.454(3) Å, *b* = 11.3549(15) Å, *c* = 9.4708(12) Å, *α* = 90.00°, *β* = 95.087(13)°, *γ* = 90.00°, and V = 2512.3(5) Å^3^. The lattice parameters at RT (293 K) were *a* = 23.948(4) Å, *b* = 11.4452(17) Å, *c* = 9.5311(15) Å, *α* = 90.00°, *β* = 96.123(14), *γ* = 90.00°, and V = 2597.5(7) Å^3^ (Table 1). Thus, we can see from the changes in compound **1** with temperature that, although the space group remained unchanged, its lattice parameter a increased by 2.10%, b increased by 0.80%, c increased by 0.64%, *β* increased by 1.09%, and volume V increased by 3.40%, indicating the presence of an isostructural phase transition in compound **1** within the range from LT to RT.

**Figure 1:**
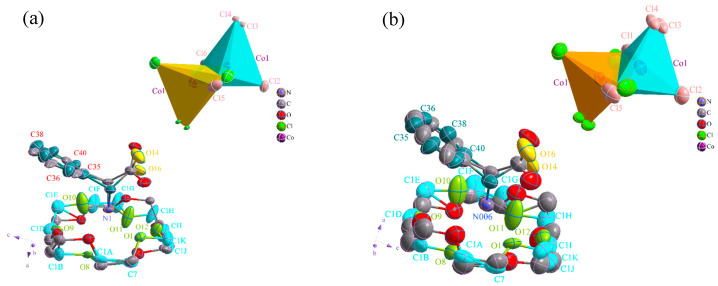
Structure chart of compound **1** with ellipsoidal probability of 50%: (**a**) low temperature (100 K); (**b**) room temperature (293 K).

Figure 1 shows the structure of compound **1** at LT (a) and RT (b), both of which share the same structural composition. The figure shows a disordered protonated DL-α-phenylglycine molecule, a disordered 18-crown-6 molecule, and a disordered [(CoCl_4_)_0.5_]^−^ anion, indicating that at certain temperatures, the 18-crown-6 and DL-α-phenylglycine molecules both undergo rocking or twisting. The occupancy ratio of carbon atoms C1A, C1B, C1D, C1E, C1F, C1G, C1H, C1I, C1J, C1K, and C7 in 18-crown-6 was 0.522 (LT) and 0.471 (RT), while the occupancy ratio of oxygen atoms O8, O9, O10, O11, O12, and O1 was 0.522 (LT) and 0.471 (RT). The occupancy ratio of benzene ring carbon atoms C35, C36, C38, and C40 in DL-α-phenylglycine was 0.522 (LT) and 0.529 (RT), while Co^2+^ exhibited a four-coordinate tetrahedral structure. Owing to the disorder of the metal skeleton, one anion [(CoCl_4_)_0.5_]^−^ appeared as two 1/2 disordered metal anion groups (CoCl_4_)^2−^. The Co-Cl bond lengths of the (CoCl_4_)^2−^ anions ranged from 2.110 to 2.291 Å (LT) and 2.173 to 2.300 Å (RT), the bond angles of Cl-Co-Cl ranged from 98.80 to 118.80° (LT) and 100.20 to 117.70° (RT), the bond lengths of the adjacent C-O ranged from 1.210 to 1.310 Å (LT) and 1.159 to 1.321 Å (RT), and the bond lengths of O-C-C ranged from 113.00 to 122.00° (LT) and 110.00 to 123.80° (RT). Thus, it can be seen that the bond lengths and bond angles changed significantly at RT owing to the thermal motion of the molecules, which can easily lead to changes in the overall structure of compound **1**, thereby resulting in anomalies in the physical properties associated with the crystal.

**Figure 2:**
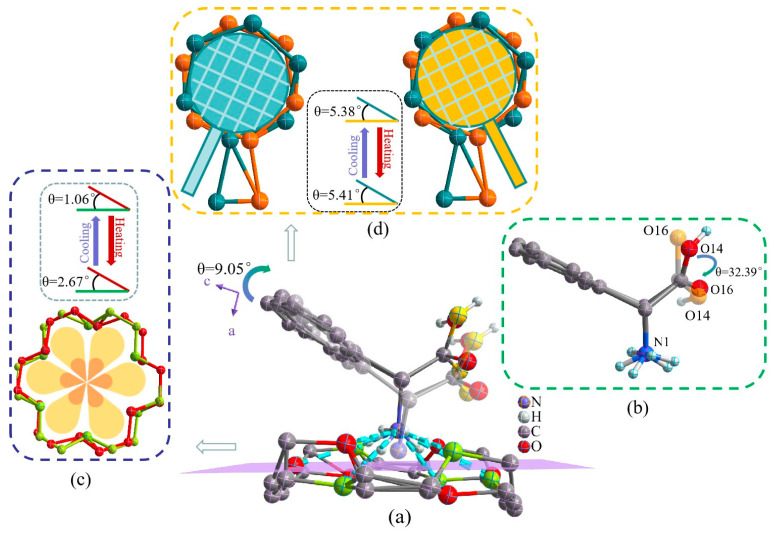
Rocking of the supramolecular cationic structure in compound **1** (**a**), rocking of the carboxyl group in protonated DL-α-phenylglycine (**b**), and twisting of the 18-crown-6 and benzene ring structures at 100 K and 293 K (**c**,**d**).

As seen in Figure 2a, the -NH_3_^+^ group in the protonated organic DL-α-phenylglycine molecule and O atom in the 18-crown-6 molecule are embedded in the cavity of cyclic 18-crown-6 through intermolecular interactions such as intermolecular N-H···O hydrogen bonds, forming an umbrella-like stator–rotor supramolecular cationic inclusion compound. At both LT and RT, the N-H···O hydrogen bonds varied slightly in bond length and bond angle, with average bond lengths of 2.932 Å (LT) and 2.980 Å (RT) and average bond angles of 125.79° (LT) and 129.68° (RT) (Table S1), which suggest that the umbrella-like supramolecular cationic inclusions were relatively strong. When the temperature increased to 293 K, the benzene ring molecules of the rotor organic component embedded in the cavity of 18-crown-6 showed a molecular rocking amplitude of 9.05° relative to the 18-crown-6 molecular plane, which was the result of steric hindrance and atomic thermal motion (Figure 2a). Figure 2b shows that the (-OH) group on the carboxyl (-COOH) group of the protonated organic component underwent significant dynamic rocking relative to the benzene ring plane, with a rocking magnitude of 32.39°. Given that disorder occurred in the structure of compound **1** at both LT and RT, the 18-crown-6 molecule underwent twisting in a structure similar to a six-petal flower, with O2–O7 and O8–O12 as the reference planes, respectively, and a twisting amplitude of 1.61° (Figure 2c). This suggests that the 18-crown-6 molecule underwent twisting from LT to RT. In addition, the disordered benzene ring molecules underwent rocking in a structure similar to a “badminton racket”, with C35–C40 and C2; C38, C43, C41, and C009; and C012 as the reference planes, respectively. The rocking amplitudes were 5.38° and 5.41° at LT and RT, respectively (Figure 2d and Figure S1). The structure shows that the microstructure of compound **1** underwent corresponding molecular twisting or rocking with the changes in temperature. The dynamic rocking of the DL-α-phenylglycine and 18-crown-6 molecules and intramolecular hydroxyl groups obtained from structural analysis are consistent with the results of variable-temperature infrared spectroscopy, thus indicating that the series of molecular rocking within the stator–rotor-type supramolecular inclusion can easily give rise to anomalies in the thermal and electrical properties of the material.

**Figure 3:**
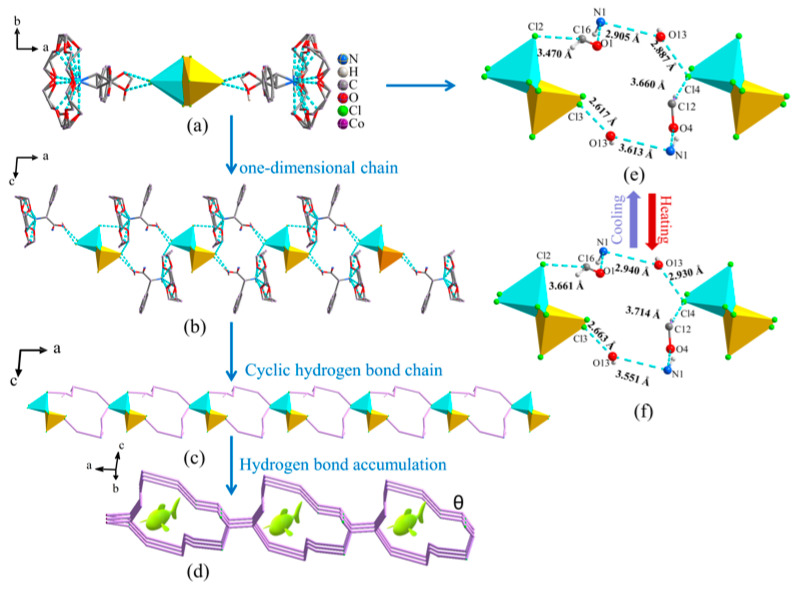
Simplest hydrogen bond diagram of compound **1** (**a**), one-dimensional hydrogen bond chain (**b**), cyclic hydrogen bond chain diagram (**c**), accumulation diagram of hydrogen bonds (**d**), and variation diagram of inorganic hydrogen bond skeleton (**e**,**f**): (**e**) low temperature (100 K); (**f**) room temperature (293 K).

As shown in Figure 3a, the supramolecular inclusion compound (DL-α-phenylglycine) (18-crown-6) forms a head-to-head linkage with the metal anion [CoCl_4_]^2−^ through O-H···Cl hydrogen bonding, and using the inorganic anion [CoCl_4_]^2−^ as a bridge, it presents an inclined, infinitely extended, double-layer, one-dimensional hydrogen bond chain alternating vertically along the *ac* plane formed via N-H···O, O-H···Cl, and C-H···Cl hydrogen bonding and intermolecular interaction forces (Figure 3b). Figure 3c shows an infinitely extended ring structure formed by hydrogen bond interactions. The anions and cations in the structure achieve a “fishnet” accumulation structure at LT and RT by twisting the cyclic hydrogen bonds on the *ac* plane, modifying the hydrogen bond C16-H16A···Cl2 from 3.470 Å (LT) to 3.661 Å (RT), N1-H1A···O13 from 3.470 Å (LT) to 3.661 Å (RT), N1-H1A···O1 from 2.905 Å (LT) to 2.940 Å (RT), O13-H13A···Cl4 from 2.887 Å (LT) to 2.930 Å (RT), and C12-H12A···Cl4 from 3.660 Å (LT) to 3.714 Å (RT) (Figure 3d). These results indicate that when triggered by temperature, the series of molecular dynamic rocking of disordered supramolecular cations will cause significant stretching motions in the one-dimensional hydrogen bond chains and hydrogen bond networks in space, thereby easily affecting the physical properties of the material.

**Figure 4:**
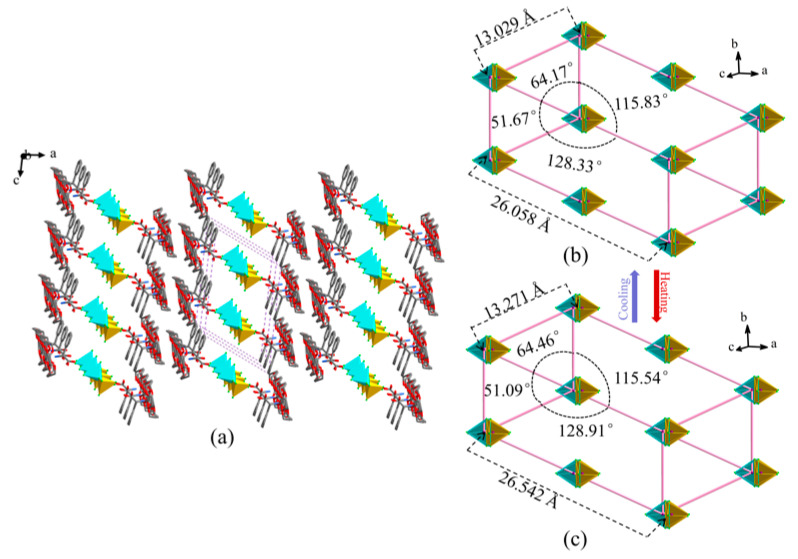
Total arrangement and accumulation diagram of compound **1** (**a**) and hexahedral structure variation with cobalt of [(CoCl_4_)_0.5_]^−^ as the vertex (**b**,**c**): (**b**) low temperature (100 K); (**c**) room temperature (293 K).

The adjacent supramolecular cations and inorganic anions formed a sandwich structure neatly arranged as bolts on the *ab* plane, as shown in Figure 4a. The inorganic anions filled the interlacing supramolecular structure and were periodically arranged in the plane to form an inclined quadrilateral net-like accumulation diagram. A regular hexahedron was obtained by taking ten adjacent [(CoCl_4_)_0.5_]^−^ along different axes as fixed points on the hexahedron, and omitting the 18-crown-6 and protonated DL-α-phenylglycine cations (Figure 4b,c). As the temperature changed, the included angles of the *cb*, *ab*, and *ca* planes and the stretching along the *a*-axis of the hexahedral structure underwent significant changes, with the interlayer spacing of the cobalt atoms increasing by 1.86%. In addition, changes occurred in both the bond lengths and bond angles, causing the deformation of the hexahedron with [(CoCl_4_)_0.5_]^−^ as the vertex. This resulted in hole stretching and intermolecular rocking in the accumulation structure, leading to changes in the electrical properties of the material.

### 2.2. Infrared and Variable-Temperature Infrared Spectroscopy of Compound ***1***

**Figure 5:**
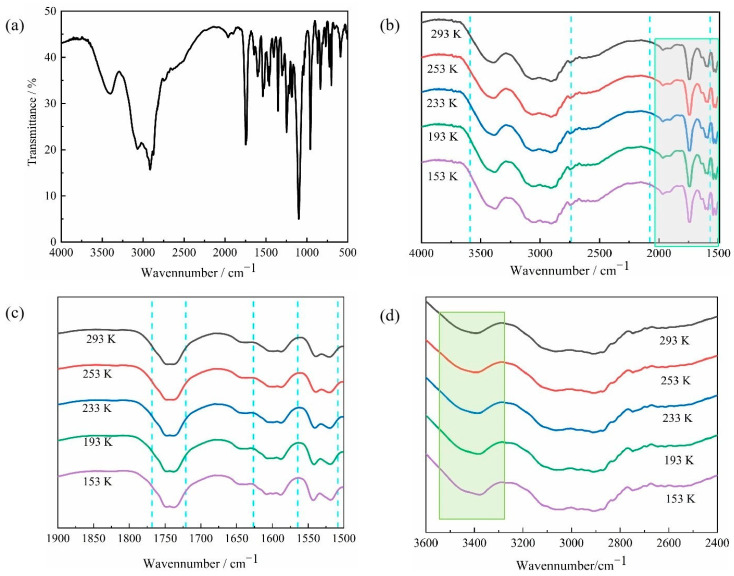
IR (**a**) and variable-temperature IR (**b**–**d**) of compound **1**.

Compound **1** was tested with infrared spectroscopy using the KBr pellet method in the 4000–400 cm^−1^ range; the results are shown in Figure 5a. Analysis of the characteristic peaks in the IR spectra showed that the peak at 3435–3375 cm^−1^ can be attributed to the stretching vibration of the -OH group; the characteristic absorption peaks at 3072–2907 cm^−1^ and 1741 cm^−1^ can be attributed to the stretching vibration of the -NH_3_^+^ and C=O groups on protonated DL-α-phenylglycine; the absorption peaks at 1108 cm^−1^, 961 cm^−1^, and 827 cm^−1^ can be attributed to the bending vibration of the -C-O-C- group on 18-crown-6; and the characteristic peaks in the range of 1357–1606 cm^−1^ can be attributed to the vibration of the aromatic ring skeleton. These findings suggest that compound **1** consists of two major components: DL-α-phenylglycine and 18-crown-6. To further determine the deformation within the structure, compound **1** was tested at 293 K, 253 K, 233 K, 193 K, and 153 K to obtain its variable-temperature IR spectra at different temperatures (Figure 5b). The results show that the shape of the characteristic peak for the -OH group at 3435–3375 cm^−1^ became broader when the temperature decreased below 233 K. When the temperature decreased to 193 K, this peak gradually fused with one characteristic peak of -NH_3_^+^ at 3190 cm^−1^ to form a broad peak, while the sharp peaks with different intensities of the C=O group at 1741 cm^−1^ underwent gradual changes with the decrease in temperature, eventually showing similar intensities and a peak shape at 193 K (Figure 5c). When the temperature decreased from 293 K to 233 K, the shape of the -OH peak remained semi-circular; however, the -OH peak became the wide type in the temperature range of 233 K–153 K (Figure 5d). The C=O group of the -COOH group found on the DL-α-phenylglycine structure of compound **1** was shown to be sensitive to changes in the molecular environment and contained hydrogen bonds formed by the -OH group. As the external temperature changed, the dipole moment increased, resulting in increasing intensity in the absorption peak and the broadening of the spectral band. The results of the variable-temperature infrared spectroscopy are consistent with the dynamic rocking of the DL-α-phenylglycine molecule and intramolecular -OH groups revealed by the crystal structure analysis, which demonstrates its structural phase transition and transformation of physical properties.

### 2.3. XRD and TG Analysis of Compound ***1***

**Figure 6:**
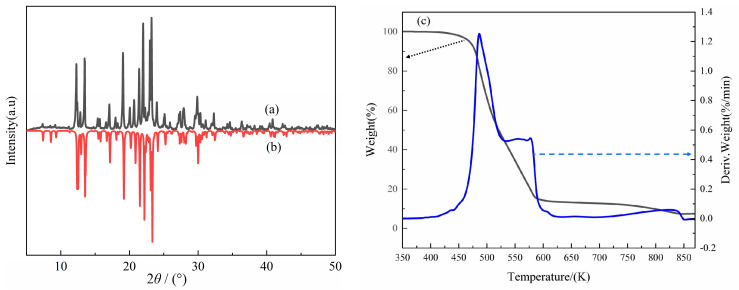
XRD pattern (**a**), simulated XRD pattern (**b**), and TG curve (**c**) of compound **1**.

The powder sample of compound **1** was tested at room temperature using a powder X-ray diffractometer in the 2*θ* angle range of 10–50°. Figure 6a shows the XRD diffractogram of compound **1** at RT (293 K), and Figure 6b shows the XRD spectrum of compound **1** obtained by simulating the single-crystal structure at RT. A comparison of both figures shows that the peak positions of the experimental and simulated data are in good agreement. Therefore, compound **1** is a single pure-phase crystal, and the test data of powder single-crystal X-ray diffraction are consistent with the crystal structure.

The TG test of compound **1** was performed in a temperature range of 350–870 K under nitrogen protection with a heating rate of 10 K/min. The results are shown in Figure 6c. No decomposition occurred in compound **1** in the range of 350–445 K, indicating that it is relatively stable in this temperature range. The TG curves show that the mass loss of compound **1** can be divided into two stages. The first stage occurred within the range of 445–586 K, with a mass decomposition ratio of 85.33%, which is generally consistent with a theoretical weight loss value of 89% for one molecule of 18-crown-6 and one molecule of protonated DL-α-phenylglycine and [Cl_4_]^−^. The second stage occurred within the range of 586–841 K, with a mass decomposition ratio of 9.30%, which is generally in agreement with the theoretical weight loss of metal oxides in compound **1**. This indicates that the components of thermal decomposition in compound **1** are consistent with the crystal structure, and that compound **1** is a crystalline material with good thermal stability.

### 2.4. Determination of Dielectric Properties

**Figure 7:**
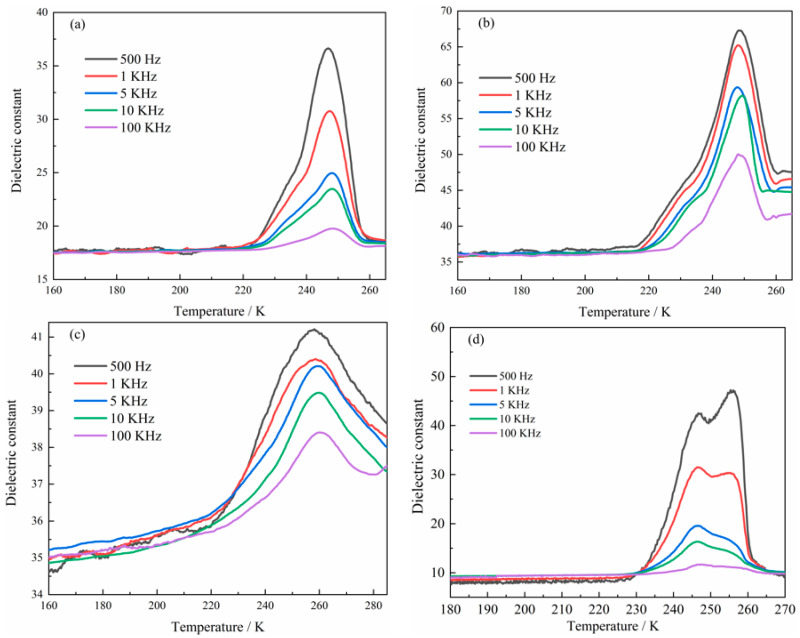
(**a**–**d**) Variations in the dielectric constant of compound **1** with temperature in different crystal axis directions: (**a**–**c**) dielectric constant for compound **1** in different crystal axis directions varying with temperature; (**d**) changes in dielectric constant of compound **1** with different temperature in tablet form.

Crystals of suitable size were selected. The three axes *a*, *b*, and *c* of the crystals were determined via single-crystal X-ray diffraction, and the crystals were tested in the form of pellets. Given that dielectric crystals tend to possess high resistivity, the three axes and crystal pellets were transformed into capacitors using conductive silver glue and copper wire. Temperature rise–variable frequency dielectric tests were carried out in the temperature range of 160 K–285 K and frequency range of 500 Hz–100 KHz. Figure 7a–d show the temperature rise dielectric constant curves of compound **1** in the three axial directions (*a*, *b*, and *c*) and in pellet form, respectively. When the temperature ranged between 160 K and 230 K and the frequency ranged between 500 Hz and 100 KHz, the dielectric constant of compound **1** in axes *a* and *b* and in pellet form tended towards a smooth linear form without significant changes. When the temperature in axes *a* and *b* exceeded 220 K, the dielectric constants showed a steep upward trend, with evident peak-like dielectric anomalies at approximately 245 K. When the temperature exceeded approximately 250 K, the dielectric constant of compound **1** decreased rapidly with the increase in temperature and eventually levelled off. Regarding the *c*-axis direction, the dielectric constant increased gradually from approximately 160 K to 220 K within the frequency range of 500 Hz–100 KHz, and when the temperature reached approximately 260 K, a clear dielectric anomaly peak appeared, followed by a gradual decrease in the dielectric constant. Concerning the dielectric constant in pellet form, it increased sharply after the temperature exceeded 230 K. When the temperature reached 245 K–255 K within a frequency range of 500 Hz–1 KHz, two consecutive peak-like dielectric anomalies appeared, and the appearance of a single peak-like dielectric constant was most evident in the frequency range of 5 KHz–100 KHz. This phenomenon was due to the mixed dielectric peaks of different axes. When the temperature reached approximately 260 K, the dielectric constant decreased sharply and eventually levelled off (Figure 7d). Combined with the structural analysis of compound **1**, the dielectric anomaly may arise from the rocking of the disordered organic DL-α-phenylglycine molecules, stretching between the one-dimensional chain structures, and deformation of the network structure with the changes in temperature, thus indicating that the compound is a dielectric anomaly-type functional material.

## 3. Experimental Section

### 3.1. Experimental Materials and Instruments

Reagents: DL-α-phenylglycine and 18-crown-6 were purchased from TCI (Shanghai, China) Development Co., Ltd. Cobalt chloride was purchased from Shanghai Macklin Biochemical Co., Ltd. (Shanghai, China) Hydrochloric acid (36.5% by mass) was purchased from Tianjin Zhiyuan Chemical Reagent Co., Ltd. (Tianjin, China). All reagents were of analytical grade, and ultrapure water was used.

Instruments: a Nicolet IS5 Fourier-transform infrared spectrometer (FTIR, Thermo Fisher Scientific, Waltham, CA, USA); Bruker SMART APEX II single-crystal X-ray diffractometer (SXRD, Bruker, Mannheim, Germany); BrukerD2 PHASER powder X-ray diffractometer (PXRD, Bruker, Mannheim, Germany, radiation source Cu-Kα, λ = 1.54056 Å, working voltage 40 kV, current 150 mA, scanning range 10°–50°); Q50 thermogravimetric analyzer (TG, TA Instruments, New Castle, DE, USA); TH2828 dielectric tester (Changzhou Tong Hui Company, Changzhou, China); and Vario E1 Cube Elemental Analyzer (Elementar, Langenselbold, Germany).

### 3.2. Synthesis of the Compound

**Figure 8:**
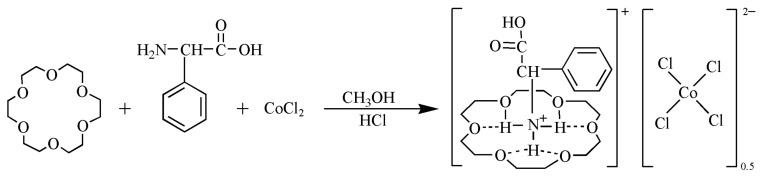
Synthesis of compound **1**.

The synthetic route of the compound [(DL-α-Phenylglycine)(18-crown-6)]^+^[(CoCl_4_)_0.5_]^−^(**1**) is shown in Figure 8. It was prepared using 10 mL of the following substances: DL-α-phenylglycine (0.114 mg, 0.756 mmol) in aqueous methanol, 18-crown-6 (0.200 mg, 0.756 mmol) in aqueous methanol, and CoCl_2_ (0.180 mg, 0.756 mmol) in aqueous methanol. The aqueous methanol solutions of CoCl_2_ and DL-α-phenylglycine were first acidified by adding 1.0 mL of concentrated hydrochloric acid (36.5% by mass). Subsequently, the aqueous methanol solutions of 18-crown-6 and CoCl_2_ were added dropwise to the aqueous methanol solution of DL-α-phenylglycine, mixed thoroughly, and left to evaporate naturally at room temperature for 10 days. A blue crystal, compound **1**, was obtained with a yield of 85%. Elemental analysis (%) C_39_H_68_C_l4_CoNO_16_ was performed: theoretical value C 46.48, N 1.39, H 6.80; experimental value C 46.21, N 1.43, H 6.38.

### 3.3. Crystal Structure of the Compound

Transparent single crystals of a suitable size (0.14 mm × 0.13 mm × 0.12 mm) and free of surface impurities were selected and loaded on a Bruker SMART APEX II single-crystal X-ray diffractometer. Using Mo-Kα radiation (λ = 0.071073 nm) with a graphite monochromator as the diffraction source, the diffraction data for compound **1** were collected at a low temperature (100 K) and room temperature (293 K). The crystal structure was analyzed based on the direct method using the SHELXL-97 program and refined based on the full matrix method in *F*^2^. Anisotropic correction was applied to all non-hydrogen atoms and the isotropic thermal parameter method was used to correct hydrogen atoms. Table 1 shows the crystallographic data of compound **1**.

## 4. Conclusions

A new crystalline material, [(DL-α-Phenylglycine)(18-crown-6)]^+^[(CoCl_4_)_0.5_]^−^(**1**), was obtained via slow evaporation using DL-α-phenylglycine, 18-crown-6, cobalt chloride (CoCl_2_), and hydrochloric acid in a methanol solution at room temperature. We tested and analyzed its structure and properties. A supramolecular stator–rotor-type inclusion compound was formed between DL-α-phenylglycine and 18-crown-6 via N-H···O hydrogen bonding compound **1**. Owing to the steric molecular rocking generated by the DL-α-phenylglycine molecule in its supramolecular cation, as well as the one-dimensional chain structure that can undergo stretching vibration on the *ac* plane formed by the inorganic anion [(CoCl_4_)_0.5_]^−^ and the supramolecular cation via hydrogen bonds O-H···Cl and C-H···Cl, the accumulation network structure within the crystal underwent changes, which resulted in a significant dielectric anomaly in compound **1** at around 245 K, 255 K, and 260 K, thereby showing significant anisotropy. Our findings indicate that compound **1** is a novel dielectric anisotropic material with potential applications in areas such as storage elements and sensors.

## Figures and Tables

**Table 1:** Crystallographic data of compound **1**.

Temperature	100 K	293 K
Chemical formula	C_39_H_68_C_l4_CoNO_16_	C_39_H_68_C_l4_CoNO_16_
Formula weight	1033.69	1033.69
Crystal size (mm^3^)	0.14 × 0.13 × 0.12	0.14 × 0.13 × 0.12
Crystal system	monoclinic	monoclinic
Space group	*C*_2_	*C*_2_
*a*/Å	23.454(3)	23.948(4)
*b*/Å	11.3549(15)	11.4452(17)
*c*/Å	9.4708(12)	9.5311(15)
*α*/(°)	90.00	90.00
*β*/(°)	95.087(13)	96.123(14)
*γ*/(°)	90.00	90.00
V/Å^3^	2512.3(5)	2597.5(7)
Z	2	2
Dc/(g·cm^−1^)	1.366	1.322
F(000)	1090	1090
μ/(mm^−1^)	0.620	0.599
2*θ* range/(°)	0.998–25.025	0.995–25.027
Rint	0.0830	0.0674
*R*^1^ [I > 2σ(I)] ^a^	0.1248	0.0805
w*R*^2^ (all data) ^b^	0.2520	0.2098
GOF	1.044	1.093

^a^ *R* = ∑(|*F*_o_| − |*F*_c_|)/∑|*F*_o_|; ^b^ w*R* = [∑*w*(|*F*_o_|^2^ − |*F*_c_|^2^)^2^/∑*w*(*F*_o_^2^)]^1/2^.

## Data Availability

Access to the corresponding author data can be provided.

## Supplementary Materials

The following supporting information can be downloaded at: https://www.mdpi.com/article/10.3390/molecules28227586/s1, Table S1: Hydrogen bond parameters of compound **1**; Figure S1: Twist diagram of the benzene ring structure at 100 K and 293 K.
