# Supplementary material for: The Synthesis, Structure, and Dielectric Properties of a One-Dimensional Hydrogen-Bonded DL-α-Phenylglycine Supramolecular Crown-Ether-Based Inclusion Compound
Source: Molecules. 2023 Nov 14;28(22):7586. doi: 10.3390/molecules28227586 (PMC10673173; doi:10.3390/molecules28227586)
Supplement: Supplementary file 1 [file molecules-28-07586-s001.zip › molecules-2675380-supplementary.pdf]

The Synthesis, Structure, and Dielectric Properties of a One-Dimensional Hydrogen-Bonded DL-α-Phenylglycine Supramolecular Crown-Ether-Based Inclusion Compound

Yang Liu [1,2,3], Hongzhi Hu [1], Huanhuan Qi [1], Meixia Lv [1] and Zunqi Liu [1,2,3,*]

Table S1. Hydrogen bond parameters of compound **1**

D—H···A	d(D—H) / Å	d(H···A) / Å	d(D···A) / Å	∠ DHA / (°)
		**1-100K**		
N1—H1BH···O8	0.890	2.100	2.967	164.700
N1—H1BH···O1	0.890	2.645	2.905	97.980
N1—H1BG···O9	0.890	2.661	2.971	101.587
N1—H1BG···O10	0.890	1.979	2.838	161.591
N1—H1BF···O12	0.890	2.005	2.863	161.672
N1—H1BF···O11	0.890	2.431	2.949	117.497
N1—H1AD···O3	0.890	2.341	2.393	123.876
N1—H1AD···O2	0.890	2.123	2.960	156.316
N1—H1AC···O4	0.890	2.245	2.878	129.997
N1—H1AC···O5	0.890	2.147	2.890	140.567
N1—H1AE···O7	0.890	2.287	2.943	130.446
N1—H1AE···O6	0.890	2.311	2.977	131.603
N1—H1AC···O1	0.890	3.048	2.905	72.344
N1—H1AC···O13	0.890	3.805	3.613	70.896
O13—H13···Cl4	0.820	3.464	2.887	140.297
O13—H13···Cl3	0.820	3.146	2.617	143.901
		**1-293K**		
N006—H00F···O8	0.890	2.432	2.996	121.686
N006—H00F···O9	0.890	2.124	2.982	162.000
N006—H00E···O10	0.890	2.263	2.883	126.496
N006—H00E···O11	0.890	2.247	2.897	129.644
N006—H00D···O1	0.890	2.297	2.939	128.996
N006—H00D···O12	0.890	2.144	2.917	144.775
N006—H00C···O6	0.890	2.592	2.996	108.529
N006—H00C···O7	0.890	2.049	2.896	158.712
N006—H00A···O4	0.890	2.579	2.936	104.863
N006—H00A···O5	0.890	1.975	2.862	173.448
N006—H00B···O2	0.890	2.378	2.973	124.357
N006—H00B···O3	0.890	2.121	2.957	156.147
N006—H00A···O1	0.890	2.685	2.939	97.637
N006—H00A···O13	0.890	3.626	3.551	78.288

O13—H13···Cl4	0.820	2.170	2.930	153.997
O13—H13···Cl3	0.820	1.904	2.663	153.441

This is the hydrogen bond length bond angle table of compound **1**. The results show that **1** is mainly composed of N-H···O and O-H···Cl. It is found that the average bond length bond angles of these two hydrogen bonds at room temperature are 2.932 Å, 125.791 ° and 2.752 Å, 142.099 °, respectively. The average bond length bond angles at room temperature are 2.980 Å, 129.684 ° and 2.797 Å, 153.719 °, respectively. It shows that the physical and chemical properties of **1** will change.

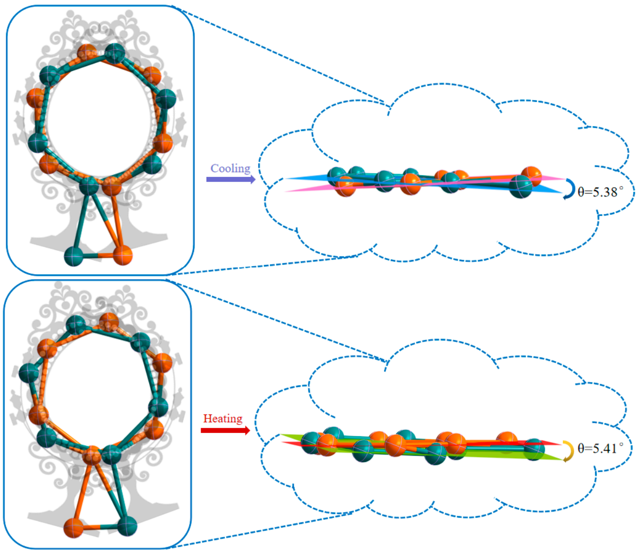

Figure S1. Twist diagram of the benzene ring structure at 100 K and 293 K

Since the structure of compound **1** is disordered at low temperature and room temperature, as shown in Fig. S1, the disordered 18-crown-6 molecules at low temperature and room temperature are based on O2~O7 and O8~O12 with a six-petal flower-like structure, respectively. The rotation amplitude is 1.61 °, indicating that the 18-crown-6 molecule rotates from low temperature to room temperature. At the same time, the disordered benzene ring molecules at low temperature and room temperature swing like a mirror, respectively, with C35~C40 and C2, C38, C43, C41, C009, C012 as the base surface swing amplitude is 5.38 °. The swing amplitude at room temperature is 5.41 °, as shown in Figure S1b. It shows that the structure of the transition compound **1** from low temperature to room temperature has undergone corresponding torsion and swing. The dynamic swing of DL-α-aminophenylacetic acid, 18-crown-6 molecule and intramolecular hydroxyl group was consistent with the results of variable temperature infrared test, indicating that a series of molecular swings in the stator-rotor supramolecular inclusion complex will be beneficial to the thermal and electrical properties of the material.
